# Cbx4 SUMOylates BRD4 to regulate the expression of inflammatory cytokines in post-traumatic osteoarthritis

**DOI:** 10.1038/s12276-024-01315-x

**Published:** 2024-10-01

**Authors:** Ding Zhou, Jia-Ming Tian, Zi Li, Jun Huang

**Affiliations:** Department of Orthopedics, The Second Xiangya Hospital, Central South University, Changsha, Hunan China

**Keywords:** Trauma, Sumoylation

## Abstract

Brominated domain protein 4 (BRD4) is a chromatin reader known to exacerbate the inflammatory response in post-traumatic osteoarthritis (PTOA) by controlling the expression of inflammatory cytokines. However, the extent to which this regulatory effect is altered after BRD4 translation remains largely unknown. In this study, we showed that the E3 SUMO protein ligase CBX4 (Cbx4) is involved in the SUMO modification of BRD4 to affect its ability to control the expression of the proinflammatory genes IL-1β, TNF-α, and IL-6 in synovial fibroblasts. Specifically, Cbx4-mediated SUMOylation of K1111 lysine residues prevents the degradation of BRD4, thereby activating the transcriptional activities of the IL-1β, TNF-α and IL-6 genes, which depend on BRD4. SUMOylated BRD4 also recruits the multifunctional methyltransferase subunit TRM112-like protein (TRMT112) to further promote the processing of proinflammatory gene transcripts to eventually increase their expression. In vivo, treatment of PTOA with a Cbx4 inhibitor in rats was comparable to treatment with BRD4 inhibitors, indicating the importance of SUMOylation in controlling BRD4 to alleviate PTOA. Overall, this study is the first to identify Cbx4 as the enzyme responsible for the SUMO modification of BRD4 and highlights the central role of the Cbx4-BRD4 axis in exacerbating PTOA from the perspective of inflammation.

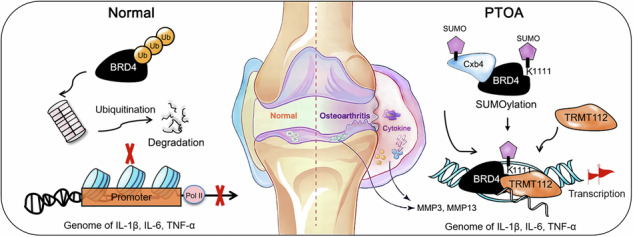

## CBX4 SUMOylation enhances BRD4 control of inflammatory genes

In research on post-traumatic osteoarthritis, scientists found a lack of knowledge on how inflammation worsens the disease. Researchers found that the protein BRD4, when altered by a process called SUMOylation, is crucial in causing inflammation in PTOA. The study used human tissue and rats to see how blocking BRD4 and a related protein, Cbx4, affects inflammation and disease development. The study involved 45 rats and examined how these proteins contribute to PTOA, using various techniques to understand their roles in inflammation. The findings showed that blocking BRD4 and Cbx4 lessened inflammation and joint damage in rats with PTOA. Specifically, treatments targeting these proteins lowered inflammation markers and improved joint health. The team concluded that the BRD4-Cbx4 interaction is a major factor in PTOA inflammation and progression, marking a significant step in understanding the disease’s molecular workings. This summary was initially drafted using artificial intelligence, then revised and fact-checked by the author.

## Introduction

Post-traumatic osteoarthritis (PTOA) is a subtype of osteoarthritis that occurs following joint injury and accounts for approximately 12% of all cases of symptomatic osteoarthritis^1^. Anterior cruciate ligament (ACL) injury, meniscus tear, glenohumeral instability, patellar dislocation, and ankle instability are the five main risk factors for PTOA, and these risk factors differ in their ways of triggering the development of osteoarthritis and the progression of the disease^2^. Trauma plays a critical role in the development of knee PTOA, with individuals who experience an injury having a more than 5-fold increased risk of developing the disease compared with the 1.7-fold relative risk associated with being overweight or obese^3–5^. Fortunately, PTOA typically has a known “starting point”, indicating that interventions can potentially be initiated at an earlier stage to prevent disease progression^6^.

A growing body of evidence shows that PTOA is exacerbated by inflammation and secondary injuries caused by initial trauma. Inflammation is a response to damage to joint cells and can lead to further degradation of cartilage and impair the repair process^7,8^. Proinflammatory cytokines such as interleukin-1β (IL-1β), interleukin-6 (IL-6), and tumor necrosis factor (TNF-α) activate specific receptors on the cell surface and intracellular signaling molecules that promote inflammation^9–12^. Therefore, interventions aimed at suppressing post-traumatic inflammation may prevent the further development of PTOA.

Bromodomain-containing protein 4 (BRD4), a member of the bromodomain and extra-terminal (BET) family, has been implicated in various diseases, including cancer, viral infections, inflammation, and neurological disorders^13^. Previous studies have shown that different inflammatory signaling pathways activated by lipopolysaccharide (LPS) converge to a rate-limiting step in the immune response, that is, converging on the transactivation of primary response genes (PRGs)^14,15^. Upon stimulation by inflammatory signals, BRD4 recruits positive transcription elongation factor b (p-TEFb) to RNA Pol II, thereby activating the transcription of PRGs^14,15^. Therefore, BRD4 is crucial in determining the extent of the inflammatory response and has also emerged as an attractive therapeutic target for blocking the inflammatory cascade in PTOA^16,17^, despite a limited understanding of its underlying mechanisms.

SUMOylation is a post-translational modification (PTM) that plays crucial roles in various biological functions, including cell growth, migration, cellular responses to stress, and tumorigenesis^18^. SUMOylation involves the attachment of small ubiquitin-like modifier (SUMO) molecules to lysine (K) residues of substrate proteins, depending on the physiological state of the cell and the specific SUMO isoform^19^. Emerging evidence indicates that SUMOylation plays a crucial role in regulating skeletal system development and homeostasis, which are closely linked to the occurrence of skeletal diseases^20^.

SUMO modification requires the involvement of SUMO E3 ubiquitin ligases to facilitate the binding of SUMO proteins to target proteins and catalyze the SUMOylation reaction. Chromobox protein homolog 4 (Cbx4), a SUMO E3 protein ligase, has been proposed as a novel therapeutic target for osteosarcoma^21^ and asthma treatment in inflammatory disorders^22,23^. In osteoarthritis, Cbx4 can counteract the aging process of human mesenchymal stem cells (hMSCs) by maintaining nucleolar homeostasis, thereby alleviating the progression of osteoarthritis^24^. In our analysis, the BRD4 protein contains multiple lysine residues that can be modified by SUMOylation, and we focused our research on this process and identified a proinflammatory mechanism of SUMOylated BRD4 that is mediated by Cbx4. These findings offer a new approach to targeted therapy for PTOA.

## Materials and methods

### Patient sample collection

Human tissue samples, including cartilage and synovial tissue samples, were obtained from male patients aged between 40 and 60 years who underwent total knee replacement surgery. Among them, the samples of the PTOA group were taken from patients with a history of previous knee trauma that progressed to severe PTOA (*n* = 5), whereas the samples of the control group were obtained from patients with recent comminuted tibial fractures due to trauma (*n* = 5). The inclusion criteria for the PTOA group included visible macroscopic pathological changes in the cartilage tissue, whereas the control group exhibited no such alterations. Additionally, the patients’ X-rays were retained, and the surgical procedure was performed by a unified team of orthopedic surgeons (Department of Orthopedics, The Second Xiangya Hospital, Central South University). Samples were obtained with the consent of all patients, and the study was approved by the Medical Ethics Committee of the Second Xiangya Hospital of Central South University with Grant No. LYF2023127.

### Animal experiments

Male Sprague‒Dawley rats (8 weeks old, 300~350 g) were obtained from JiangSu Aniphe Biolaboratory, Inc. (Nanjing, Jiangsu, China), and acclimated for 1 week at 25 ± 1 °C with a 12 h light/dark cycle. A total of 45 rats were randomly divided into four groups: the sham group, anterior cruciate ligament transection (ACLT) group, BRD4 inhibitor group (JQ1 or ARV-285), and Cbx4 inhibitor group (UNC3866), with 5 rats in each group. The implementation of ACLT surgery followed the methods outlined in our team’s previous research^25^, ensuring that the rats underwent the procedure while under anesthesia. Inhibitors of BRD4 and Cbx4 (Selleck CN, Shanghai, China) were injected into the knee cavity of the rats one week after surgery and continued for one week. The dose administered was 5 mg/mL, with each injection consisting of 50 μL. Two weeks after the end of the intervention, the weights of the rats were measured, and the Osteoarthritis Research Society International (OARSI) score was used to measure the severity of osteoarthritis in the rats from three aspects: dynamic pain, joint swelling and muscle dysfunction around the knee joint. The rats were euthanized with 5% isoflurane, and then, the knee cavity tissues were collected for analysis. The animal experiments in this study were conducted in accordance with the regulations set by the Animal Ethics Committee of the Second Xiangya Hospital of Central South University with Grant No. 2022014.

### Histopathological analysis

The pathological alterations in the knee cartilage and synovial tissues of the patients and rats were examined via Safranin O-fast green and Alcian blue staining, as described in previous studies^26^. Before histopathological analysis, the cartilage tissues were rendered soft through decalcification via an acidic solution and subsequently fixed in 4% neutral formalin, after which the resulting tissues were processed into 5 μm pathological slides for staining and microscopy image acquisition (3D-HISTECH, Budapest, Hungary) to facilitate pathological analysis.

### Primary synovial fibroblast isolation, culture, treatment, and transfection

Synovial fibroblasts were isolated from the synovium of the knee joints of the rats and were identified via methylene blue staining according to the manufacturer’s instructions (Servicebio, Wuhan, China). The cells were cultured in a 5% CO_2_ incubator at 37 °C in DMEM12 supplemented with 10% FBS (Gibco, New York, USA) and 1% penicillin/streptomycin. For generation of an inflammatory environment, 10 ng/mL HMGB1 recombinant rat protein (Abcam, Cambridge, UK) was added to the culture medium for 24 h. For plasmid transfection, the cells were initially seeded in a 6-well plate and incubated for 24 h. The expression plasmids for BRD4 or Cbx4 (pcDNA3.1 vector) or the shRNA plasmids for Cbx4 or TRMT112 (hU6 vector) were subsequently mixed with Lipofectamine 3000 (Invitrogen, California, USA) and introduced into the medium for 48 h. The targeted sites of the shRNAs used in this study are listed in Table 1.

**Table 1:** List of shRNA target sequences for genes.

Genes	Targeted sequences
BRD4 (*Rattus*)	5’-GCAGACCAACCAACTGCAATA-3'
Cbx4 (*Rattus*)	5’-GCTTCTGAGGAGAAGAAAGCC-3'
TRMT112 (*Rattus*)	5’-GGAAGATGCACCATGTGTTGC-3'

### Quantitative polymerase chain reaction (qPCR)

Total RNA was extracted from tissues or cells via the TRIzol reagent (Vazyme, Nanjing, China). Subsequently, 500 ng of each RNA sample was subjected to cDNA synthesis via a reverse transcription kit (Promega, Madison, Wisconsin, USA). SYBR Green-based qPCR was then performed on the basis of established protocols^27^. GAPDH served as an internal control for normalization purposes. The quantification of target gene expression was accomplished via the 2^−ΔΔCT^ method. The primers used were synthesized by GeneScript (Nanjing, Jiangsu, China), and the sequences are listed in Table 2.

**Table 2:** List of the qPCR primer sequences used in this study.

RNAs	Primer sequence
mBRD4 (*Rattus*)	Forward, 5’-ACAGCCCAACAGAACAAAC-3’; Reverse, 5’-GTGCTGGTTCCTTCTTGCTC-3'
mCbx4 (*Rattus*)	Forward, 5’-AGCCGGAGGTCATCCTTTTG-3’; Reverse, 5’-AAATTCACTCAGGGGCTCCG-3'
mIL-1β (*Rattus*)	Forward, 5’-CAGCAGCATCTCGACAAGAG-3’; Reverse, 5’-CATCATCCCACGAGTCACAG-3'
Pre-IL-1β (*Rattus*)	Forward, 5’-GAAGCTGTGGCAGCTACCTATG-3’; Reverse, 5’-GCACTAAGGTCTTGCACTTGG-3'
mTNF-α (*Rattus*)	Forward, 5’-GAAACACACGAGACGCTGAA-3’; Reverse, 5’-CAGTCTGGGAAGCTCTGAGG-3'
Pre-TNF-α (*Rattus*)	Forward, 5’-GCCTCAGCCTCTTCTCATTCC-3’; Reverse, 5’-CCTCCCCAACTCTCCTCCAC-3'
mIL-6 (*Rattus*)	Forward, 5’-CCACCCACAACAGACCAGTA-3’; Reverse, 5’-AACGGAACTCCAGAAGACCAG-3'
Pre-IL-6 (*Rattus*)	Forward, 5’-GGATACCACCCACAACAGACC-3’; Reverse, AGAACCCAGCAGAGAATTCCC-3'
mGAPDH (*Rattus*)	Forward, 5’-GCAAGTTCAACGGCACAG-3’; Reverse, 5’-GCCAGTAGACTCCACGACAT-3'
mBRD4 (*Homo*)	Forward, 5’-GTGGTGCACATCATCCAGT-3’; Reverse, 5’-CCGACTCTGAGGACGAGAAG-3'
mCbx4 (*Homo*)	Forward, 5’-GGTCGCCCAATATAACACG-3’; Reverse, 5’-GGTCAGGACATTGGAACGAC-3'
mIL-1β (*Homo*)	Forward, 5’-CGACACATG GGATAACGA-3’; Reverse, 5’-CGCAGGACAGGTACAGAT TC-3'
mTNF-α (*Homo*)	Forward, 5’-GGCTGCCTTGGTTCAGATGT-3’; Reverse, 5’-CAGGTGGGAGCAACCTACAGTT-3'
mIL-6 (*Homo*)	Forward, 5’-CCAACTTCCAATGCTCTCCTAATG-3’; Reverse, 5’-TTCAAGTGCTTTCAAGAGTTGGAT-3'
mGAPDH (*Homo*)	Forward, 5’- CTGACTTCAACAGCGACACC-3’; Reverse, 5’-GTGGTCCAGGGGTCTTACTC-3'

### Western blotting

In brief, the antibodies were hybridized to proteins isolated via SDS‒PAGE and transferred to polyvinylidene fluoride membranes (Millipore, Massachusetts, USA). Primary antibodies against BRD4 (AiFang Biological, Hunan, China, 1:500), Cbx4 (Abcam, 1:500), IL-1β (Abcam, 1:1000), TNF-α (Abcam, 1:1000), IL-6 (Abcam, 1:1000), MMP3 (Merck, Darmstadt, Germany, 1:1000), MMP13 (Merck, 1:1000), SUMO1 (Abcam, 1:1000), SUMO2/3 (Merck, 1:1000), TRMT112 (CST, Boston, USA, 1:1000) and HA (Abcam, 1:2000) were diluted to a working concentration and incubated overnight at 4 °C, followed by incubation with the corresponding horseradish peroxidase (HRP)-conjugated secondary antibodies (CST, 1:3000) at room temperature for 1 h, after which signals were detected via chemiluminescence via a ChemiDoc system (Bio-Rad, Munich, Germany). The data were finally analyzed via ImageJ software V1.8.0 (National Institutes of Health, Maryland, USA).

### Immunohistochemistry (IHC)

The paraffin-embedded sections were subjected to antigen retrieval. After being blocked with 10% goat serum, the slides were incubated overnight with BRD4 (AiFang Biological, 1:200), Cbx4 (Abcam, 1:200), IL-1β (Abcam, 1:500), TNF-α (Abcam, 1:500), and IL-6 (Abcam, 1:500) primary antibodies at 4 °C and HRP-conjugated secondary (CST, 1:1000) antibodies at 25 °C for 1 h. The signals generated were visualized via a mouse- and rabbit-specific HRP/AEC IHC detection kit (AiFang Biological).

### Coimmunoprecipitation (co-IP) analysis

In the immunoprecipitation procedure, IP-grade antibodies specific for the target proteins were employed, with the execution of particular methodologies derived from the author’s prior expertise^27,28^. The antibodies utilized for co-IP analysis were as follows: BRD4 (CST, 1:50), Cbx4 (CST, 1:50), SUMO1 (Abcam, 1:100), SUMO2/3 (Merck, 1:100), and HA (CST, 1:100).

### Assessing the severity of rat knee osteoarthritis through micro-CT evaluation

As previously described, all the rats were subjected to a well-established surgical model to induce OA, followed by intra-articular injection of specific inhibitors as required by the study protocol. Two weeks post-intervention, the rats were euthanized under excessive anesthesia, and the knee joint cavities were dissected. High-resolution imaging techniques utilizing microcomputed tomography (micro-CT) were subsequently employed to obtain volumetric data on the knee joint. The obtained images were then processed through CTvox software (Bruker micro-CT, Massachusetts, USA) for three-dimensional reconstruction and segmentation, allowing a detailed analysis of joint tissue structure and changes such as bone destruction and cartilage damage.

### Chromatin immunoprecipitation (ChIP) sequencing and bioinformatics analysis of the global effects of BRD4 inhibition on potential target genes

The male SD rats (8-week-old, weighing 300~350 g) were grouped into three categories: sham, ACLT, and ACLT + (+)−JQ1. The rats initially underwent ACLT surgery to induce knee joint trauma, followed by intra-articular injection of the BRD4 inhibitor (+)−JQ1 (5 mg/mL, 50 μL). After 6–8 h, the rats were euthanized, and their knees were dissected. The skin and muscle surrounding the knee joints were removed, and the entire knee joint, including the articular cartilage, subchondral bone of the femur and tibia, patella, meniscus, ligaments, synovium, and joint capsule tissues, was obtained by cutting at the distal femoral growth plate and proximal tibial growth plate. A portion of the harvested tissues was frozen in liquid nitrogen and pulverized via a mortar and pestle under liquid nitrogen for BRD4 expression detection. Another portion was fixed with formaldehyde and used for the BRD4 ChIP experiment. The immunoprecipitated DNA products were then sent to the Beijing Genomics Institute (Shenzhen, China) for genome sequencing, and subsequent analysis included Gene Ontology (GO) and Kyoto Encyclopedia of Genes and Genomes (KEGG) enrichment analysis of differentially binding genes.

### Detection of RNA Pol II or H3K27ac binding to the promoter regions of the IL-1β, TNF-α, and IL-6 genes via a ChIP assay

Tissue or cell samples fixed with paraformaldehyde were lysed and mechanically disrupted via ultrasonication to generate chromatin fragments with sizes ranging from 200 to 500 bps. The resulting fragments were subjected to immunoprecipitation following the instructions provided by the manufacturer of the kit (CST, Cat. No. 9003). For this process, either the RNA pol II or H3K27ac ChIP grade antibody (CST, 1:100) was utilized. The genomic products precipitated by the ChIP antibodies were analyzed by qPCR to amplify the promoter fragments of IL-1β, TNF-α, and IL-6. The obtained data are presented as a percentage (%) of the input.

### RNA pulldown

The pre-mRNA sequences of IL-1β, TNF-α, and IL-6 were synthesized, and the sense and antisense RNA probes were biotinylated (Sangon Biotech, Shanghai, China). These probes were obtained through in vitro transcription via the MESSAGE mMACHINE® Kit (Invitrogen, California, USA) with the target RNA sequence and template. The cellular samples were subsequently lysed and mixed with biotin-labeled RNA probes and streptavidin magnetic beads (Thermo Fisher Scientific, MA, USA) to form RNA‒protein complexes. Finally, the beads were washed, and the resulting products were subjected to western blot analysis to determine the presence of the target proteins BRD4 or TRMT112.

### Measurement of mRNA stability

For evaluation of mRNA stability, transcription was inhibited by the administration of actinomycin D (Selleck CN, 5 µg/mL) for 0, 2, and 4 h. Reverse transcription was then conducted using an equal amount of RNA across all time intervals, and the mRNA abundance was quantified via qPCR.

### Statistical analysis

The results are presented as the mean ± standard deviation (SD) unless otherwise stated. For the comparison of two groups, statistical significance was determined via two-tailed unpaired Student’s *t* tests. For comparisons among multiple groups, one-way analysis of variance (ANOVA) with Tukey’s or Dunnett’s post hoc test was conducted. All figures were generated via GraphPad Prism 9 software (GraphPad Software, Inc., San Diego, California, USA). A *P* value < 0.05 was considered statistically significant for all experiments, and the significance in all figures is indicated as follows: *P < 0.05, **P < 0.01, ***P < 0.001.

## Results

### The SUMOylation of BRD4 is elevated in the synovium of patients diagnosed with PTOA

**Fig. 1:**
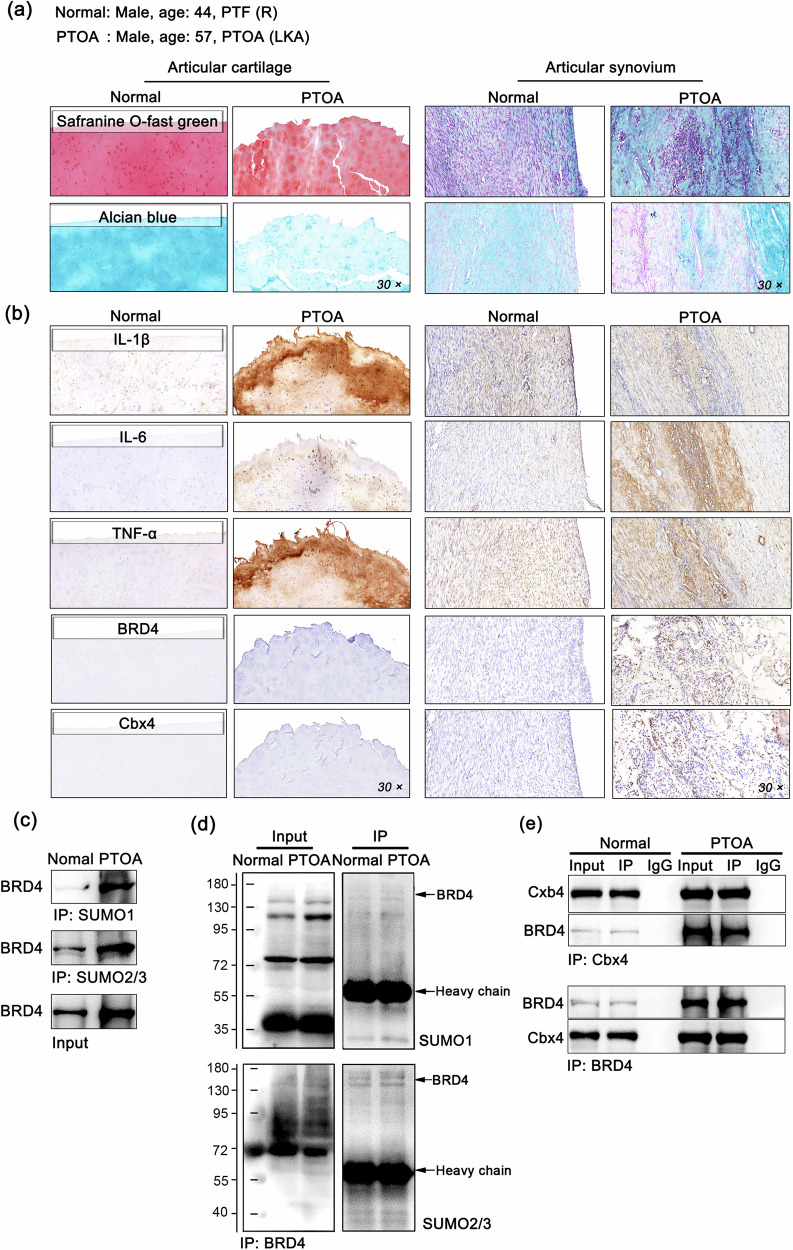
The SUMOylation of BRD4 is elevated in the synovium of patients diagnosed with PTOA. **a** Representative images of Safranin O-fast green and Alcian blue staining of cartilage and synovial tissues from 2 patients. PTF (R): comminuted fracture of the tibial plateau (right); KOA (L): knee osteoarthritis (left); **b** The protein levels of IL-1β, TNF-α, IL-6, BRD4, and Cbx4 in patient articular cartilage and synovial tissues determined via IHC. **c** The level of SUMO-modified BRD4 determined via co-IP in the immunoprecipitates of SUMO1 and SUMO2/3 from patient articular synovial tissues. **d** The levels of SUMO1 and SUMO2/3 in BRD4 immunoprecipitates from patients’ articular synovial tissues were determined via co-IP, which revealed the presence of SUMO-modified BRD4 products. **e** The endogenous binding relationship between BRD4 and Cbx4 in patient synovial tissues was determined via co-IP. In **c**–**e**, mixed samples within the group, *n* = 5.

Tissue sections of the patient knee cartilage and synovial tissue samples were examined with Safranin O-fast green and Alcian blue staining to assess the extent of cartilage cells, the cartilage matrix, and fibrous tissue lesions. The results indicated that patients with PTOA presented rough surfaces with severe fibrosis or hypertrophic chondrocyte changes (Fig. 1a). The expression levels of the inflammatory proteins IL-1β, TNF-α and IL-6 in the cartilage and synovial tissue of the PTOA patients were significantly greater than those in the controls. Notably, BRD4 or Cbx4 expression was not detected in healthy or diseased cartilage tissues, but significant upregulation was observed in the synovium of the patients with PTOA (Fig. 1b). In addition, BRD4 exhibited increased SUMOylation levels in the synovial tissues of the PTOA patients compared with the healthy controls (Fig. 1c, d). Furthermore, there was a significant interaction between Cbx4 and BRD4 in the synovial tissues of the PTOA patients (Fig. 1e), suggesting that Cbx4 may be involved in the SUMO modification of BRD4 and that alterations in this process could lead to changes in the biological functions of BRD4 during the inflammatory pathological process.

### The SUMOylation of BRD4 is elevated in the synovium of rats with knee arthritis induced by ACLT

**Fig. 2:**
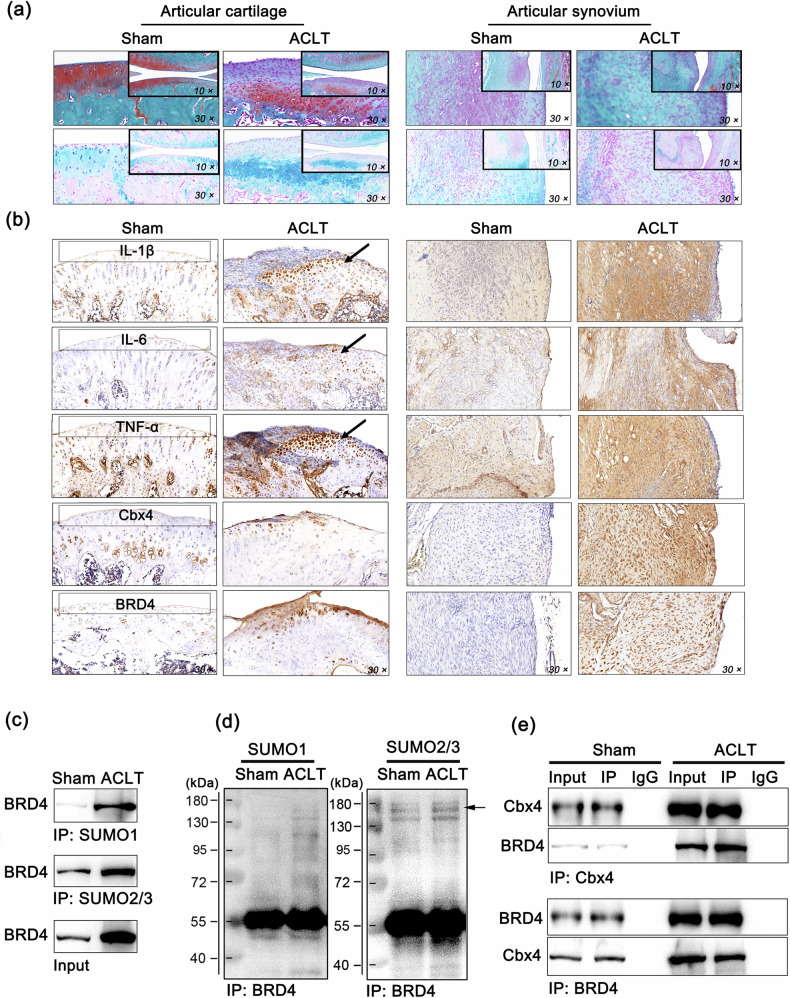
The SUMOylation of BRD4 is elevated in the synovium of rats with knee arthritis induced by ACLT. **a** Representative images of Safranin O-fast green and Alcian blue staining of cartilage and synovial tissues from rats. **b** The protein levels of IL-1β, TNF-α, IL-6, BRD4, and Cbx4 in rat articular cartilage and synovial tissues were determined via IHC. The black arrows indicate areas of significantly increased expression. **c** The level of SUMO-modified BRD4 determined via co-IP in the immunoprecipitates of SUMO1 and SUMO2/3 from rat synovial tissues. **d** The levels of SUMO1 and SUMO2/3 in BRD4 immunoprecipitates from rat synovial tissues were determined via co-IP, which revealed the presence of SUMO-modified BRD4 products. **e** The endogenous binding relationship between BRD4 and Cbx4 in rat synovial tissues was determined via co-IP. In **c**–**e**, mixed samples within the group, *n* = 5. Drug dosage, frequency, and duration of use: (+)−JQ1 (5 mg/mL, 50 μL) and ARV-285 (5 mg/mL, 50 μL) were administered once daily for one week.

Given that the incidence of PTOA after ACL injury is as high as 87%^29^, the ACLT method was then adopted in this study to establish a rat PTOA model. Similarly, Safranin O-fast green and Alcian blue staining were used to examine the chondrocyte, cartilage matrix, and fiber composition in cartilage tissues of the knee joint. The ACLT procedure clearly caused severe injury to the cartilage tissue of the rats, characterized by fibrosis on the cartilage surface; abnormal proliferation of the chondrocytes in each layer; and significant increases in the levels of the inflammatory cytokines IL-1β, TNF-α, and IL-6 (Fig. 2a, b). Consistent with the clinical observations, negligible expression of BRD4 or Cbx4 was observed in the knee cartilage of the rats, but BRD4 or Cbx4 was significantly expressed in the synovial tissue of the model group (Fig. 2b). In addition, there was a substantial increase in the SUMOylation of BRD4 in synovial tissues from the model group, with a strengthened interaction between BRD4 and Cbx4 (Fig. 2c–e). Overall, we found that the SUMOylation of BRD4 was elevated in the synovial tissues of PTOA patients and ACLT model rats.

### BRD4 inhibitors alleviate ACLT-induced knee arthritis in rats

**Fig. 3:**
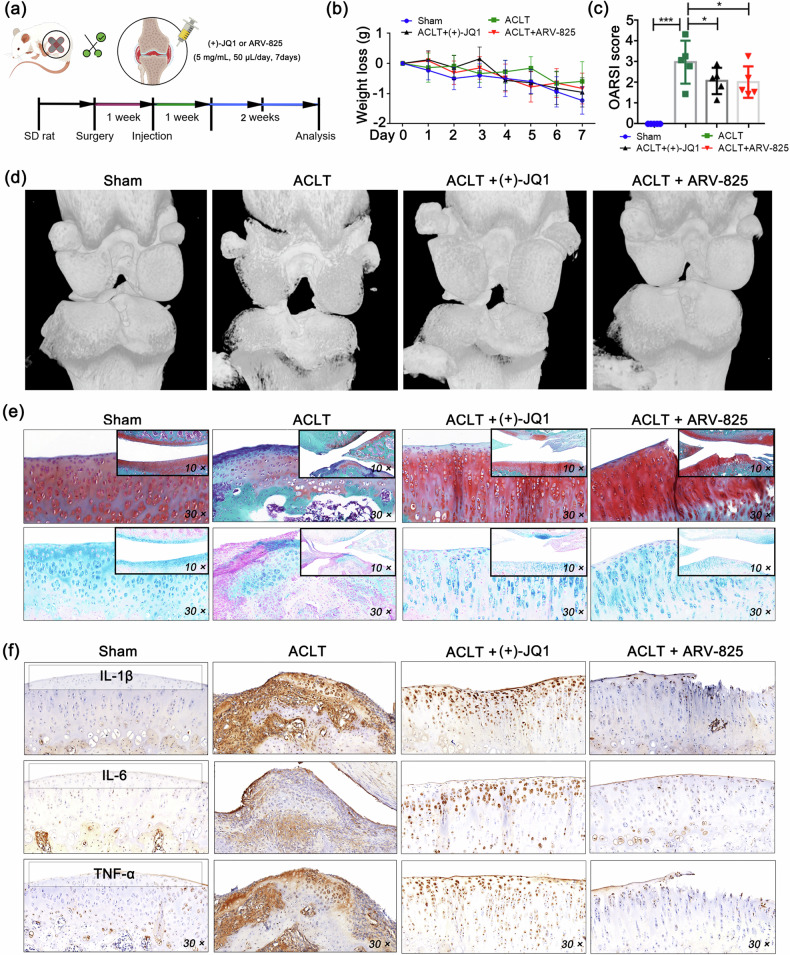
BRD4 inhibitors alleviate ACLT-induced knee arthritis in rats. **a** Schematic diagram of the animal experimental procedure. **b** Rat weight loss data. **c** OARSI scores of the rats. **d** Three-dimensional imaging of rat knee tissues via micro-CT scanning. **e** Representative images of Safranin O-fast green and Alcian blue staining in the cartilage tissues of the rats. **f** The protein levels of IL-1β, TNF-α, IL-6, BRD4, and Cbx4 in the cartilage tissues of the rats were determined via IHC. *N* = 5, one-way ANOVA. *P < 0.05; **P < 0.01; ***P < 0.005.

BRD4 is a well-established target for intervention in diseases such as cancer and immune-related inflammation^30–32^, so the development of BRD4 inhibitors has reached a relatively advanced stage. Given that BRD4 is promoted in ACLT model rats, we used BRD4 inhibitors, (+)−JQ1 and ARV-825, for postmodeling intervention to determine whether either of them could ameliorate the inflammatory phenotype. As a competitive inhibitor of BRD4, (+)−JQ1 impedes BRD4’s epigenetic reading function by displacing BRD4 from acetylated chromatin^33^, whereas ARV-825 effectively degrades the BRD4 protein by recruiting BRD4 to the E3 ubiquitin ligase^34^. The results of the animal experiments revealed that there was no significant weight loss in the rats within 7 days of intra-articular injection of BRD4 inhibitors, indicating the relative safety of the inhibitor intervention (Fig. 3a, b). The OARSI scores demonstrated that BRD4 inhibition effectively alleviated arthritis in the rats (Fig. 3c). Moreover, histopathological analysis revealed an increase in the cartilage matrix of the knee, a decrease in fibrosis, and a significant reduction in the levels of the inflammatory factors IL-1β, TNF-α, and IL-6 in the cartilage in the intervention group compared with those in the ACLT model group (Fig. 3d–f). These findings indicate a notable alleviation of joint arthritis in the rats treated with BRD4 inhibitors.

**Fig. 4:**
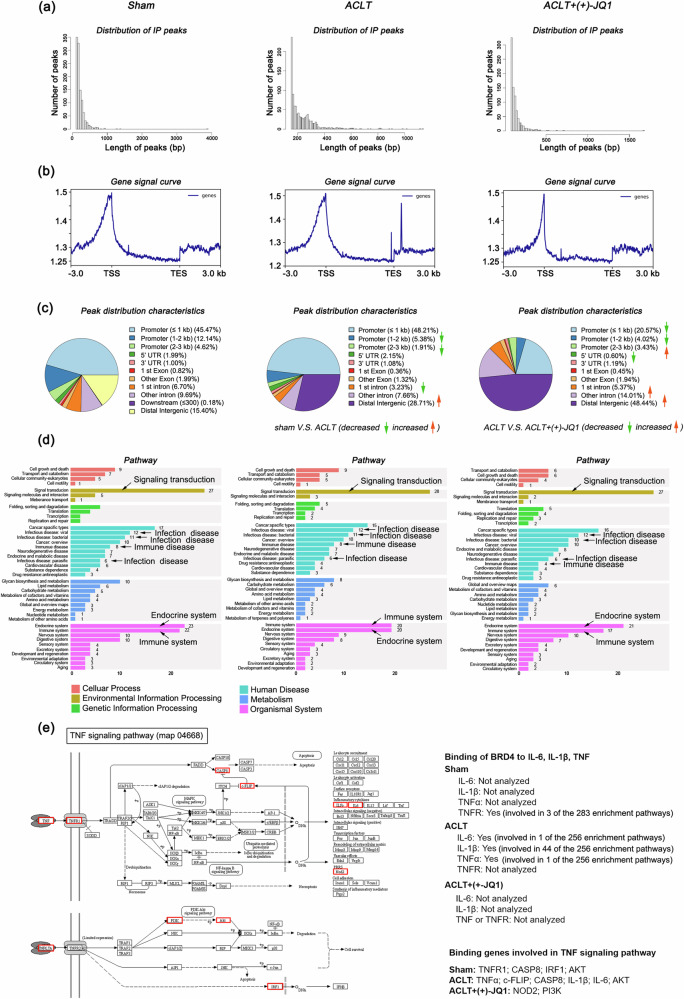
Analysis of BRD4-binding chromatin information in rat knee tissue. **a** ChIP-seq assay for BRD4 was used to analyze the chromatin distribution of BRD4 in the knee tissues of the rats in each group. **a** Length distribution of the peaks. **b** Gene signal curve. **c** Peak distribution characteristics of genetic elements for each group. **d** Secondary classification of the KEGG pathway analysis results. **e** Brd4-bound genes involved in TNF signaling pathways (KEGG pathway map04668).

Given that BRD4 functions as a chromatin reading protein that typically acts as a transcriptional cofactor to aid in the activation of downstream target genes^35^, we conducted a global analysis of the chromatin information of BRD4 binding in rat knee joint tissues via a ChIP-seq assay (Fig. 4a, b). Compared with those of the sham-operated group, the binding level of BRD4 in the promoter region (within 0–3 kb) of the knee joint tissues of the rats subjected to ACLT was lower (sham: 62.23%, ACLT: 55.50%), with the main decrease occurring within 1–3 kb of the binding region (sham: 16.76%, ACLT: 7.29%), while there was a slight increase in binding abundance within 1 kb (sham: 45.47%, ACLT: 48.21%). Conversely, the ACLT group presented a significant increase in the binding level of BRD4 in the intergenic regions (sham: 15.40%, ACLT: 28.71%). Furthermore, compared with that in the ACLT model group, the binding level of BRD4 in the inhibitor-treated group decreased to half of the original level (ACLT: 55.50%, ACLT + (+)−JQ1: 28.02%). However, in the intergenic regions, the binding level of BRD4 increased significantly from 28.71% in the ACLT group to 48.44% in the inhibitor-treated group (Fig. 4c). These findings indicate that the inhibitor effectively inhibits BRD4 and competitively displaces BRD4 from the promoter region of the gene, thereby suppressing gene transcription.

Further investigation into the KEGG pathways enriched by genes bound to BRD4 revealed that a majority of these genes are associated with signal transduction, as indicated by the analysis of environmental information processing. An analysis of human disease revealed that in addition to participating in the cancer pathway, most of the genes bound to BRD4 are associated with infectious and immune-related diseases. Additionally, analysis of the organismal system revealed that the genes bound to BRD4 are related primarily to the immune system and endocrine system (Fig. 4d), indicating the important role of BRD4 in endocrine function^36^ and immune function^37^ in bone tissue. Notably, in the knee joint tissues of the rats in the sham group, BRD4 did not bind to the IL-1β, TNF-α, or IL-6 gene locus, although TNF receptor 1 (TNFR1) may be regulated by BRD4. However, in the ACLT group, BRD4 was found to bind to the genes encoding IL-1β, TNF-α, and IL-6, particularly IL-1β, which participated in 44 of the 256 enriched pathways, whereas TNF-α and IL-6 were involved in the TNF signaling pathway (KEGG map04668). In the knee joint tissues of the rats treated with (+)−JQ1, no gene fragments of IL-1β, TNF-α/TNFR1, or IL-6 were detected in the chromatin immunoprecipitation products of BRD4 (Fig. 4e). These findings suggest that in the rats subjected to ACLT, inflammation mediated by BRD4 is ongoing and sustained in knee joint tissues, whereas after the inhibition of BRD4, the transcription of inflammatory cytokines may be controlled. All BRD4 target genes involved in the TNF signaling pathway that were identified by ChIP-Seq analysis are as follows: TNFα, TNFR1, IL-1β, IL-6, caspase 8, interferon regulatory factor 1 (IRF1), phosphatidylinositol 3-kinase (PI3K), RAC-alpha serine/threonine-protein kinase (AKT), cellular FLICE-inhibitory protein (C-FLIP), and nucleotide-binding oligomerization domain-containing protein 2 (NOD2) (Fig. 4e).

**Fig. 5:**
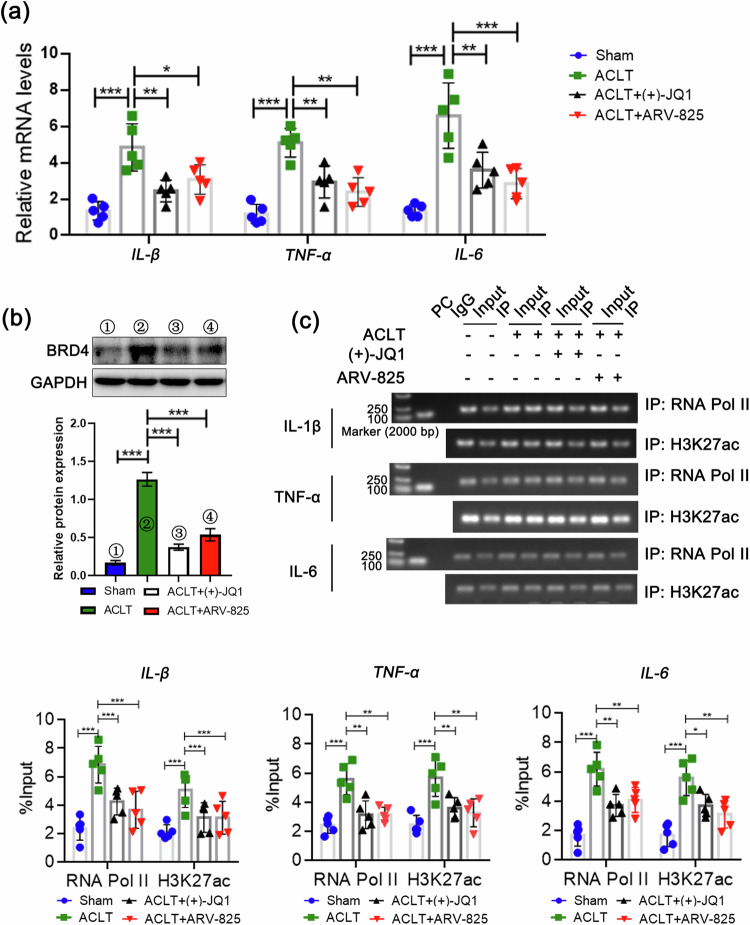
BRD4 inhibitors reduce the transcriptional activity of inflammatory cytokine genes in the synovial tissue of rats. **a** Relative mRNA expression of IL-1β, TNF-α and IL-6 in synovial tissues of rats determined by qPCR. **b** Relative protein expression of BRD4 determined by western blotting. **c** The degree of enrichment of H3K27ac and RNA Pol II in the promoter region was used as indicators of the transcriptional activity of genes, and ChIP/qPCR was used to evaluate the transcriptional activity of IL-1β, TNF-α and IL-6. n = 5; one-way ANOVA. *P < 0.05; **P < 0.01; ***P < 0.005.

Given that the reduced binding of BRD4 to the promoter region may result in a weakened interaction between the promoter and enhancer^38^, this phenomenon could lead to a decrease in the recognition and binding of transcription factors and RNA Pol II to the promoter regions, consequently limiting gene transcription levels. Therefore, we examined the relative mRNA expression levels of IL-1β, TNF-α, and IL-6 in synovium and analyzed the binding abundance of RNA Pol II and H3K27ac in the promoter regions of the IL-1β, TNF-α, and IL-6 genes to assess whether BRD4 affects their transcriptional activity. When BRD4 was suppressed by BRD4 inhibitors, the transcriptional activities of IL-1β, TNF-α, and IL-6 were significantly diminished (Fig. 5a–c). These findings demonstrate the good anti-inflammatory effect of targeting BRD4 in alleviating ACLT-induced joint arthritis in rats.

### HMGB1/TLR4 signaling induces BRD4-dependent IL-1β, TNF-α and IL-6 transcription in synovioblasts

**Fig. 6:**
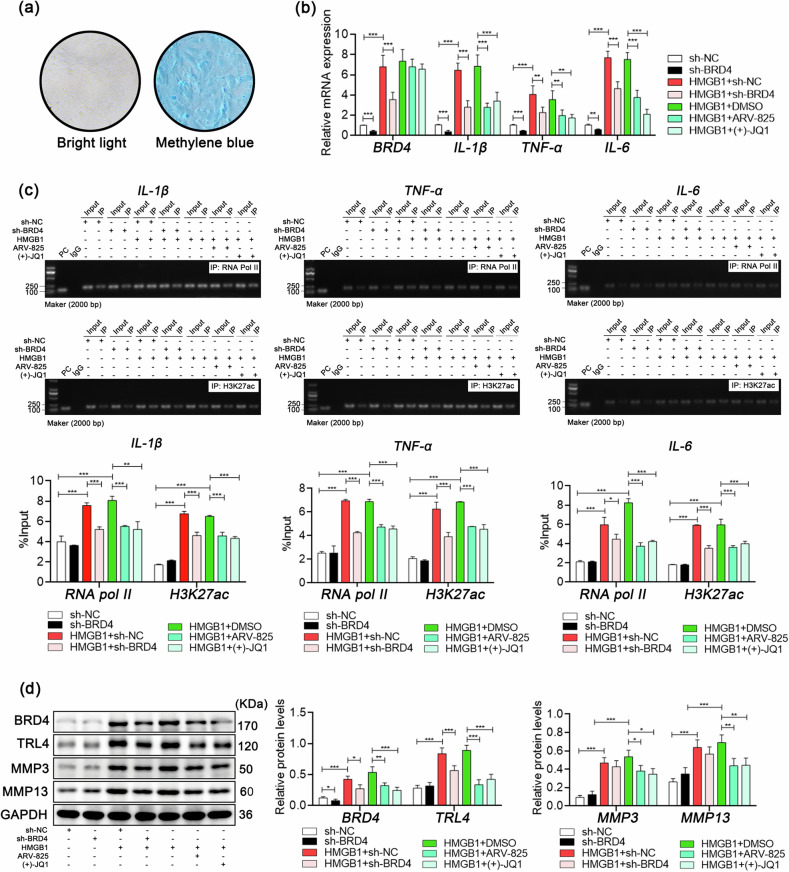
Inflammation induces BRD4-dependent IL-1β, TNF-α and IL-6 transcriptional activity in synovial fibroblasts. **a** Primary isolated rat joint synovial fibroblasts stained with methylene blue. **b** Relative expression levels of BRD4, IL-1β, TNF-α, and IL-6 mRNA in synovial fibroblasts determined by qPCR. **c** Transcriptional activities of IL-1β, TNF-α and IL-6 in synovial fibroblasts evaluated by ChIP/qPCR. **d** Relative protein expression levels in synovial fibroblasts determined by western blotting. Drug dosages and durations: HMGB1 (10 ng/mL), (+)−JQ1 (100 nM), and ARV-285 (100 nM) for 24 hours. In **b**–**d**, *n* = 3, one-way ANOVA. *P < 0.05; **P < 0.01; ***P < 0.005.

High mobility group box 1 (HMGB1) simulates the activation of the innate immune system in the absence of foreign invaders by directly binding to its membrane receptor Toll-like receptor 4 (TLR4)^39^, so we chose this molecule to stimulate the inflammatory response of synovial cells cultured in vitro. First, synovial fibroblasts were isolated from the rat synovium (Fig. 6a), followed by the addition of HMGB1 to induce an inflammatory response. BRD4 inhibition was subsequently performed in cells via shRNA interference or targeted inhibitors, (+)−JQ1 and ARV-825, according to research needs. The results revealed that the protein expression of TLR4 and BRD4 in synovial fibroblasts significantly increased after HMGB1 treatment, as did the transcriptional activities of the IL-1β, TNF-α, and IL-6 genes. Notably, any intervention inhibiting BRD4 significantly suppressed these changes induced by HMGB1 (Fig. 6b, c). In addition, BRD4 inhibition effectively controlled the protein expression of the matrix metalloproteinases MMP3 and MMP13 in synovial fibroblasts (Fig. 6d). These results suggest the effectiveness of BRD4 inhibition in preventing inflammation in cells.

### HMGB1/TLR4 signaling induces Cbx4-mediated SUMOylation of BRD4

**Fig. 7:**
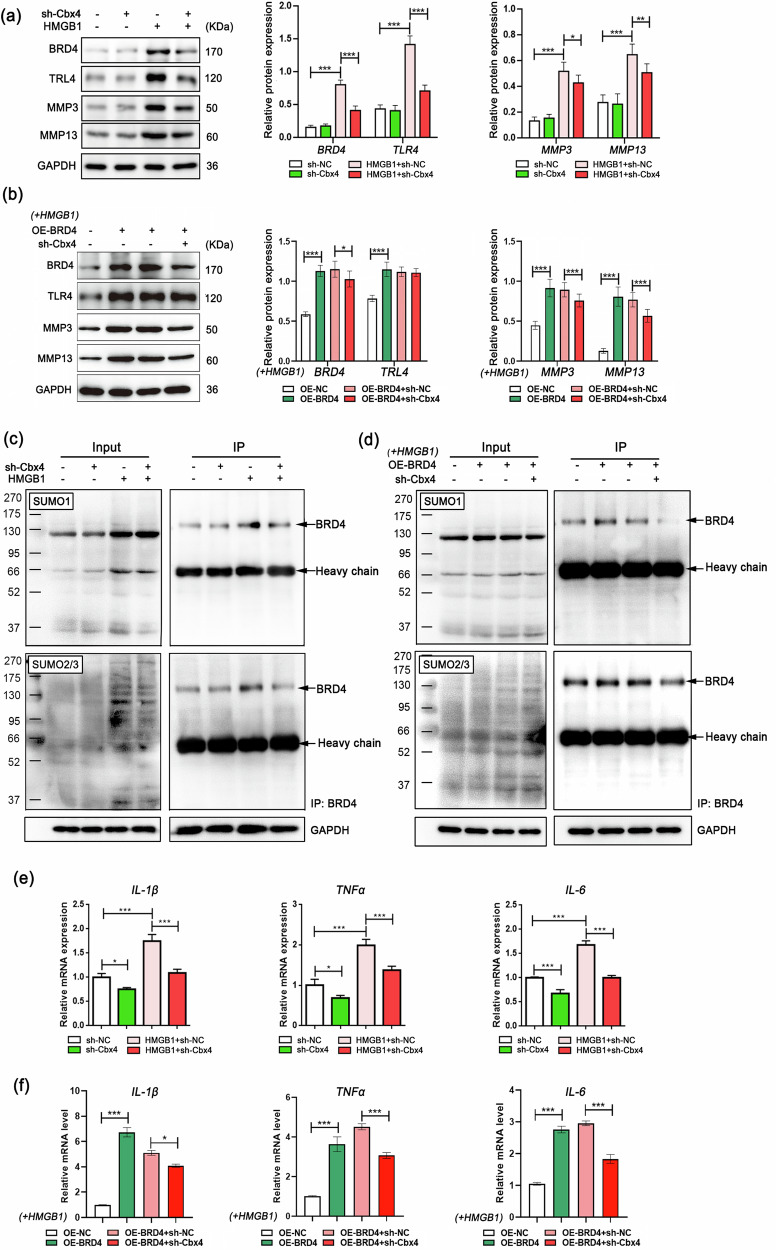
Inflammation induces Cbx4-mediated SUMOylation of BRD4. **a**, **b** Relative protein expression levels in synovial fibroblasts determined by western blotting. **c**, **d** The levels of SUMO1 and SUMO2/3 in BRD4 immunoprecipitates from rat synovial cells were determined via co-IP, which revealed the presence of SUMO-modified BRD4 products. **e**, **f** Relative mRNA expression levels of IL-1β, TNF-α and IL-6 determined by qPCR. In **a**, **b**, **e**, and **f**, *n* = 3, one-way ANOVA. *P < 0.05; **P < 0.01; ***P < 0.005.

We further investigated the importance of Cbx4 for BRD4 SUMOylation in PTOA. To this end, we constructed a synovial fibroblast model in which Cbx4 expression was silenced, which subsequently promoted inflammation through HMGB1. Inhibiting Cbx4 did not significantly affect the protein expression levels of BRD4, TLR4, MMP3, or MMP13 in cells cultured in the absence of HMGB1. However, when inflammation was triggered by HMGB1, Cbx4 inhibition was highly effective in suppressing the overall expression levels of these proteins (Fig. 7a). Moreover, the loss of Cbx4 expression resulted in a decrease in the SUMOylation level of BRD4 induced by HMGB1, accompanied by a reduction in the mRNA expression and transcriptional activities of IL-1β, TNF-α, and IL-6 induced by HMGB1/TLR4 signaling (Fig. 7c, e). In addition, silencing Cbx4 expression in synovial fibroblasts substantially reduced the SUMO-modified BRD4 protein levels and hindered the activation of the HMGB1/TLR4 signaling pathway, as well as the increased expression of inflammatory cytokines induced by BRD4 upregulation (Fig. 7b, d, f). In general, the data provided in this section support the involvement of Cbx4-mediated BRD4 SUMOylation in the regulation of inflammatory cytokine production in synovial fibroblasts.

### Cbx4 mediates the SUMOylation of BRD4 at K1111

**Fig. 8:**
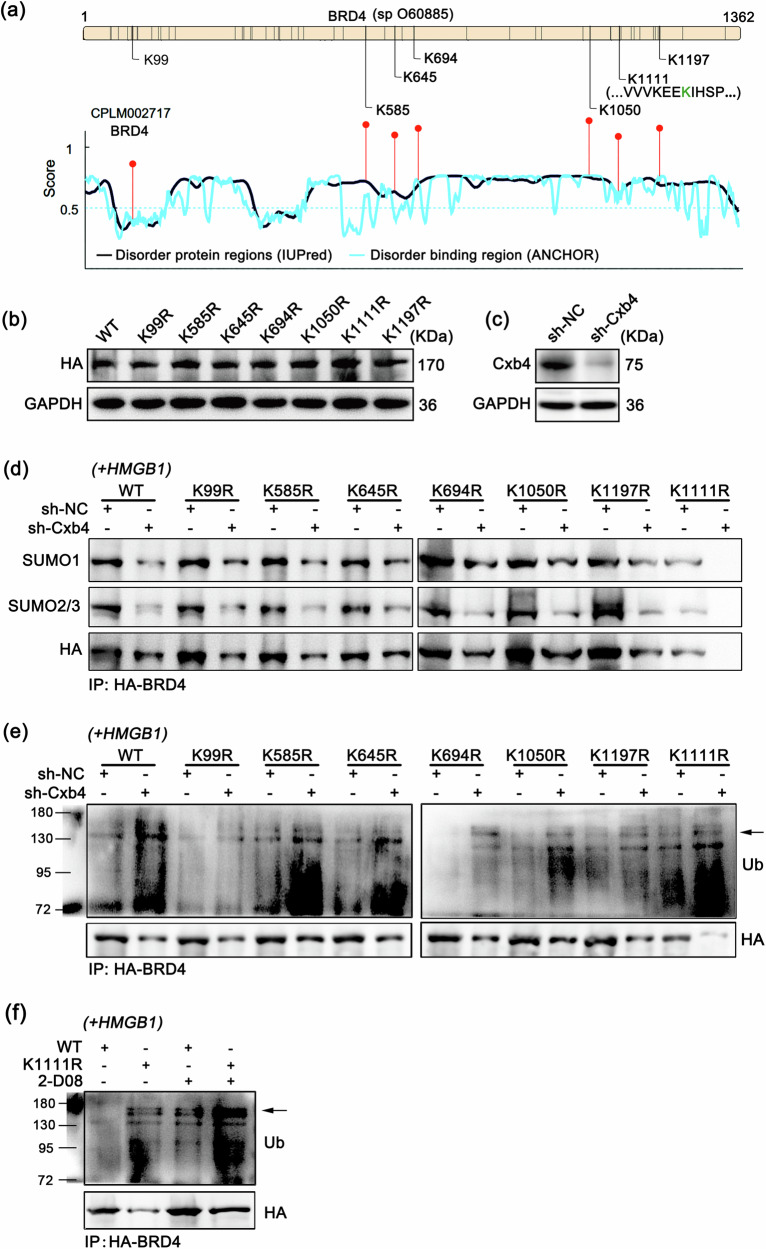
Cbx4 mediates K1111 SUMOylation of BRD4. **a** Potential SUMOylation sites in the BRD4 protein predicted via the UniProt database (https://www.uniprot.org) and the GPS-SUMO database (https://sumo.biocuckoo.cn/). **b** Relative HA expression levels in each BRD4-mutated overexpression group determined by western blotting. **c** Relative protein expression of Cbx4 in the synovial fibroblasts of the rats was determined via western blotting. **d** The protein contents of SUMO1 and SUMO2/3 in each BRD4 (wild-type or mutant) and HA-fused overexpressing cell subjected to inflammatory induction by HMGB1 were determined via co-IP with an anti-HA antibody. **e** Ubiquitination levels of the BRD4-HA fusion protein determined in the same cell samples as in **d** by the co-IP method. **f** Ubiquitination level of the BRD4-HA fusion protein determined by co-IP after 2-D08 treatment. Drug dosages and durations: HMGB1 (10 ng/mL) and 2-D08 (5 nM) for 24 h.

BRD4 possesses multiple K residues that can be modified by SUMOylation, including K99, K585, K645, K694, K1050, K1111, and K1197 (Fig. 8a). However, it remains unclear which of these sites can be regulated by Cbx4 to undergo SUMO modification. Therefore, we constructed expression vectors for BRD4 and systematically mutated the mentioned K residues to R residues while introducing an HA tag to determine the impact of Cbx4 on these potential modification sites (Fig. 8b, c). The results revealed that when Cbx4 was downregulated, the SUMO1 and SUMO2/3 levels in the HA immunoprecipitation products of BRD4 mutants decreased. In particular, in the K1111R group, SUMO1 and SUMO2/3 expression was the lowest or nearly undetectable (Fig. 8d). Additionally, the downregulation of Cbx4 led to an increase in the ubiquitination level of BRD4, with the wild-type (WT) group exhibiting the lowest level and the K1111R group exhibiting the highest level (Fig. 8e). Furthermore, we investigated the influence of SUMOylation at the K1111 site on the ubiquitination of BRD4. The findings revealed a greater level of BRD4 ubiquitination in the WT group than in the K1111R group following the administration of the SUMOylation inhibitor 2-D08 (Fig. 8f), indicating that the stability of the BRD4 protein relies on its SUMOylation, particularly at the K1111 site. Taken together, these results demonstrate that Cbx4 primarily facilitates the SUMOylation of BRD4 at the K1111 site, thus rendering it less susceptible to ubiquitination-induced degradation.

### Inhibition of Cbx4 alleviates ACLT-induced knee arthritis in rats

**Fig. 9:**
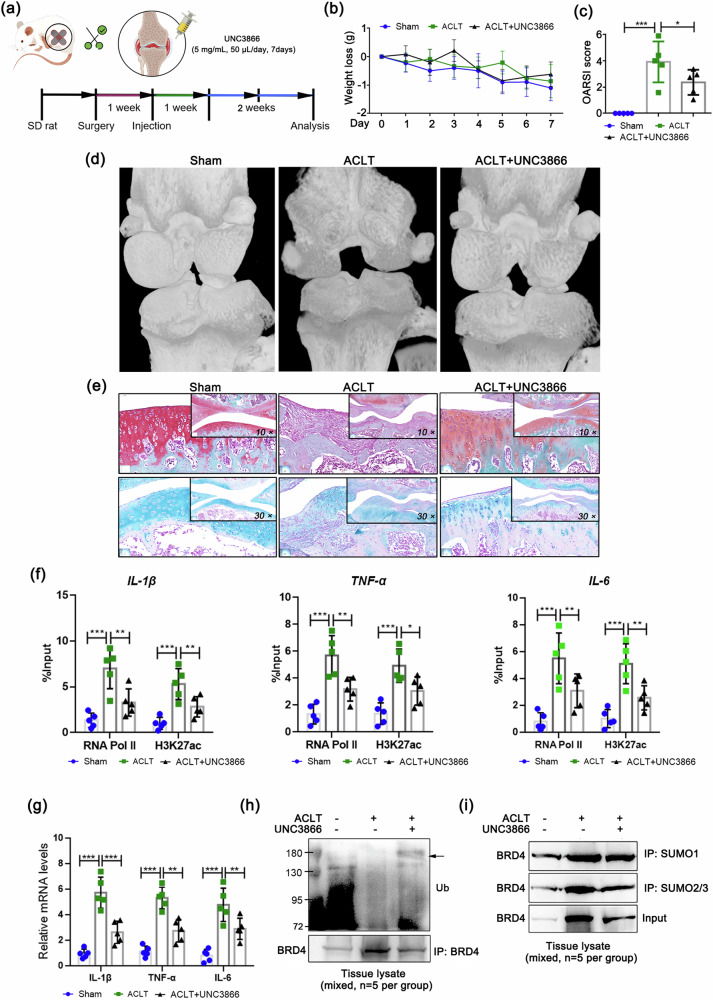
Inhibition of Cbx4 alleviates ACLT-induced knee arthritis in rats. **a** Schematic diagram of the animal experimental procedure. **b** Rat weight loss data. **c** OARSI scores of the rats. **d** Three-dimensional imaging of rat knee tissues via micro-CT scanning. **e** Representative images of Safranin O-fast green and Alcian blue staining of cartilage tissues from rats. **f** Transcriptional activities of IL-1β, TNF-α and IL-6 evaluated by ChIP/qPCR. **g** Relative mRNA expression levels of IL-1β, TNF-α and IL-6 were determined by qPCR. Ubiquitination (**h**) and SUMOylation (**i**) levels of BRD4 in the synovial tissues of rats determined by co-IP. Drug dosage, frequency, and duration of use: UNC3866 (5 mg/mL, 50 μL) was administered once daily for one week. In **c**, **f**, and **g**, *n* = 5, one-way ANOVA. *P < 0.05; **P < 0.01; ***P < 0.005.

We aimed to further investigate the potential of inhibiting Cbx4 to alleviate inflammatory characteristics in vivo and utilized a Cbx4 inhibitor (UNC3866) to model intervention in rats (Fig. 9a). There was no significant weight loss in the rats throughout the 7-day intervention period (Fig. 9b), and a notable decrease in the severity of knee arthritis was observed compared with that in the nonintervention group, as measured by OASRI scores (Fig. 9c). In addition, the pathological status of the cartilage was significantly improved in the inhibitor intervention group, manifesting as an increase in the matrix and a smoother surface (Fig. 9d, e), whereas the mRNA levels, transcriptional activities, and protein levels of the inflammatory cytokines IL-1β, TNF-α, and IL-6 in the synovium were significantly decreased (Fig. 9f, g). In terms of its effect on BRD4, Cbx4 inhibition significantly decreased SUMOylation and increased BRD4 ubiquitination in vivo (Fig. 9h, i). The results of this study indicate that inhibiting Cbx4 is also an effective strategy for combating the pathological changes and accumulation of inflammatory proteins in ACLT-induced knee arthritis in rats and that Cbx4 plays a vital role in controlling the stability of the BRD4 protein.

### BRD4 recruits TRMT112 to ensure the expression efficacy of inflammatory cytokines

**Fig. 10:**
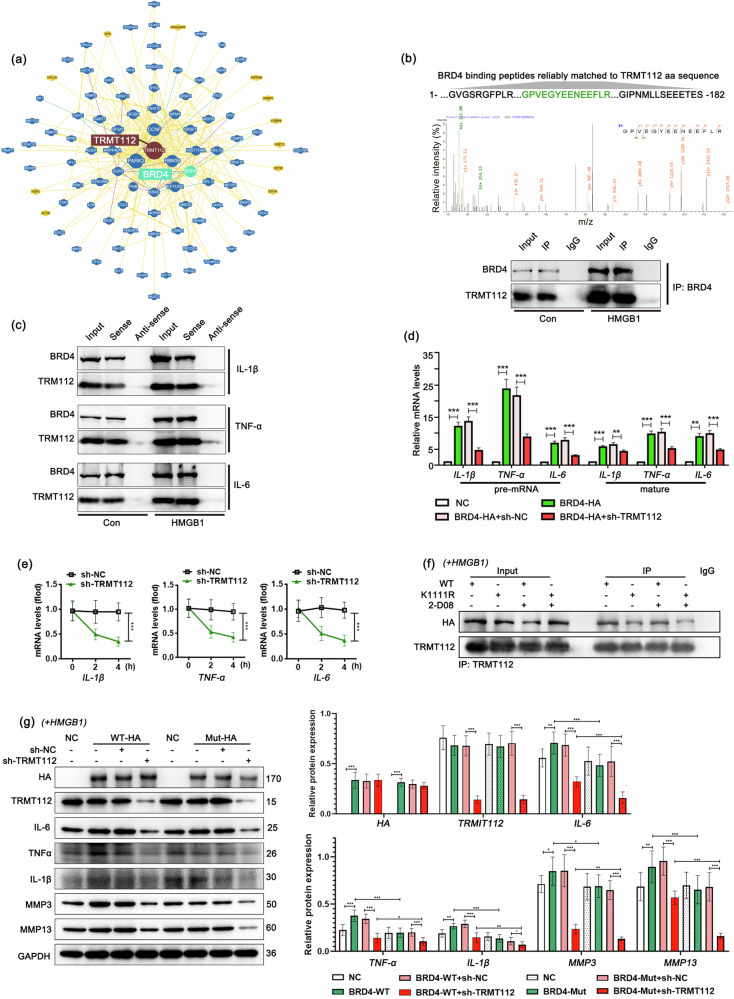
BRD4 recruits TRMT112 to ensure the expression efficacy of inflammatory cytokines. **a** Binding relationship between BRD4 and TRMT112 according to the BioGRID database (https://thebiogrid.org). **b** The peptides of the TRMT112 protein that bind to endogenous BRD4 were analyzed by BRD4 co-IP/MS, and the binding relationship between TRMT112 and BRD4 in synovial fibroblasts from rats was verified via co-IP. Green-labeled amino acid sequence: reliable matching with the TRMT112 amino acid sequence analyzed via the BRD4 co-IP/MS technique. **c** The binding of IL-1β, TNF-α and IL-6 pre-mRNAs to BRD4 or TRMT112 was determined in the synovial fibroblasts of rats via the RNA pulldown technique. **d** Relative mRNA expression levels determined by qPCR. **e** RNA stability was assessed via RNA digestibility tests. **f** The degree of TRMT112 binding to the BRD4-HA fusion protein was determined via co-IP after 2-D08 treatment and K1111R mutation. **g** Relative protein expression levels determined by western blotting. Drug dosages and durations: HMGB1 (10 ng/mL) or 2-D08 (5 nM) for 24 h. In **d** and **g**, *n* = 3, one-way ANOVA. In **e**, *n* = 3, Student’s *t* test. *P < 0.05; **P < 0.01; ***P < 0.005.

The augmenting influence of BRD4 on inflammatory cytokines may not be confined to transcriptional regulation. Among the BRD4-binding proteins assessed via mass spectrometry (MS), a multifunctional methyltransferase subunit named TRM112-like protein (TRMT112), with the peptide GPVEGYEENEEFLY that binds to BRD4, attracted our attention. TRMT112 is a small, evolutionarily conserved protein that functions as a cofactor and activator of various transmethylases (MTases) involved in rRNA, tRNA, and protein methylation^40–45^, and the binding association between BRD4 and TRMT112 led us to consider that BRD4 may also participate in the post-transcriptional regulation of target RNAs. To verify this, we confirmed the presence of endogenous binding between BRD4 and TRMT112 in HMGB1-induced synovial fibroblasts via a co-IP assay, which aligns with data from the BioGRID database (Fig. 10a, b). Moreover, we detected BRD4 and TRMT112 in the pulled-down products of the IL-1β, TNF-α, and IL-6 pre-mRNA probes, suggesting post-transcriptional regulation of these inflammatory cytokines in the BRD4-TRMT112 complex (Fig. 10c).

Next, TRMT112 was interfered with by shRNA in BRD4-overexpressing synovial fibroblasts to determine whether the RNA processing of IL-1β, TNF-α, and IL-6 was reliant on TRMT112. The results revealed that the relative expression levels of precursors and mature RNAs of IL-1β, TNF-α and IL-6 were significantly increased when BRD4 was elevated but decreased sharply when TRMT112 was suppressed (Fig. 10d). In addition, TRMT112 was shown to be essential for the stability of these inflammatory cytokine mRNAs through RNA digestibility experiments (Fig. 10e).

To investigate whether BRD4 SUMOylation affects the recruitment of TRMT112 and subsequently influences the RNA stability of inflammatory cytokines, we treated WT-HA- and K1111R-HA-overexpressing cells with 2-D08 and measured the levels of BRD4-HA in TRMT112 immunoprecipitates. The results revealed that both 2-D08 treatment and mutation of the K1111 site of BRD4 led to a decrease in the amount of BRD4 in TRMT112 immunoprecipitates (Fig. 10f). Moreover, interference with TRMT112 expression significantly counteracted the upregulation of inflammatory cytokines and matrix metalloproteinases induced by BRD4 overexpression, and this effect was more significant in cells from the K1111R group than in those from the WT overexpression group (Fig. 10g). Overall, these data demonstrate that BRD4’s inflammatory-promoting effects are partially achieved through its SUMOylation followed by a TRMT112-dependent pathway.

## Discussion

This study revealed the upregulation of Cbx4 and SUMOylated BRD4 in the synovial tissues of knee joints in patients with PTOA and in a rat model of osteoarthritis-induced ACLT. By administering inhibitors of BRD4 or Cbx4 into the joint cavity, we successfully suppressed the inflammatory response in osteoarthritis and reduced cartilage damage in rats. In cultured synovial fibroblasts, the HMGB1/TRL4 inflammatory signaling pathway induced an increase in the protein levels of Cbx4 and BRD4, which subsequently activated the transcriptional activity of genes encoding inflammatory cytokines such as IL-1β, TNF-α, and IL-6. Mechanistically, we discovered that SUMOylation of BRD4 at the K1111 site was facilitated by Cbx4, which plays a role in preventing the degradation of BRD4 via ubiquitination. Consequently, BRD4 recruits TRMT112 to ensure the processing and maturation of IL-1β, TNF-α, and IL-6 gene transcripts, which leads to more severe inflammation. Collectively, these findings underscore the importance of the Cbx4-BRD4 axis in promoting inflammation in PTOA.

BRD4 on chromatin reportedly moves during inflammation^46^, and inhibiting BRD4 recognition of acetylated histones by BET inhibitors helps prevent the overproduction of inflammatory proteins^47^. Targeting BRD4 is also beneficial for a variety of musculoskeletal disorders, such as acute rheumatoid arthritis^48^, post-traumatic osteoarthritis^49^, gouty arthritis^50^, ovariectomized osteoporosis^51^, and intervertebral disc degeneration^52^. In this study, we provide additional evidence supporting a positive role for PTOA through the inhibition of Cbx4-dependent BRD4 SUMO modifications. Notably, we did not detect significant expression of the BRD4 and Cbx4 proteins in human or rat chondrocytes. However, in inflamed synovial tissues and synovial fibroblasts, these genes were found to be significantly upregulated, which is consistent with the findings of Kerstin Klein et al.^48^. At this point, we hypothesize that this result may be because the chondrocytes in the joint cavity are not the typical inflammatory cell type, whereas synovial cells are. Synovial cells secrete inflammatory mediators and participate in cartilage destruction during the inflammatory process, which was confirmed by our follow-up experiments.

Post-translational modifications, such as glycosylation^53^, SUMOylation^54^, or methylation^55^, typically promote protein stabilization, thereby influencing various cellular processes, such as transcriptional regulation, RNA processing, translation accuracy, and protein nuclear trafficking. Conversely, ubiquitination promotes protein degradation^56^. Although the substrates of SUMOylation regulate several biological pathways, including inflammation^57,58^, drugs targeting global SUMOylation are not suitable for mitigating inflammation because of the essential role of these modifications in various biological processes, nor is there a clear correlation between global SUMOylation levels and inflammation^58^. In this study, we alleviated knee inflammation in rats by inhibiting the E3 SUMO ligase Cbx4, analyzed the major SUMOylation site of BRD4 mediated by Cbx4 (K1111) and observed that BRD4 undergoes modifications in response to inflammatory signals to protect BRD4 from ubiquitination-mediated degradation.

On the basis of our current findings, we cannot completely rule out that other K residues of BRD4 may undergo SUMOylation. However, we propose a plausible explanation that strongly supports the likelihood of SUMOylation occurring at the K1111 site of BRD4. The attachment of SUMO modifiers to proteins plays a crucial role in regulating cellular signaling pathways through noncovalent binding with SUMO-interacting motifs (SIMs), and studies have further revealed that the interaction between SUMO and SIMs can be modulated by post-translational modifications such as phosphorylation and acetylation. Specifically, phosphorylation near the SUMO modification site significantly increases the strength of the SUMO-SIM interaction, potentially up to one order of magnitude^59–62^. Moreover, acetylation of K residues has been found to act as a switch for regulating SUMO modification at this site^63^. Upon examination of potential SUMO modification sites in the amino acid sequence of BRD4, several adjacent phosphorylation sites, such as Ser1110, Ser1117, Ser1126, and Ser1128, were found to be present near the K1111 site, implying that the linkage of SUMO molecules at the K1111 site may be more stable. Furthermore, a study conducted in yeast fungi has shown that the K1111 site of BRD4 undergoes N6-acetylation^64^, which is a unique characteristic not observed in other potential SUMO modification sites. Therefore, it can be reasonably inferred that the acetylation of the K1111 site determines its potential for SUMOylation.

A comprehensive examination of the detailed mechanism underlying the inhibition of BRD4 degradation through ubiquitination caused by SUMOylation at the K1111 site is lacking. However, there have been multiple literature reports and reviews discussing the positive impact of target protein SUMOylation on protein stability. The prevailing understanding is that SUMO molecules can competitively interact with ubiquitin molecules on K residues, thereby impeding the ubiquitination process of protein substrates^65^. Additionally, SUMOylation may induce structural changes in proteins, which further affect protein‒protein interactions, subcellular localization, and stability^66^. Fully elucidating the mechanism of crosstalk between these two modifications remains a complex undertaking; nonetheless, we contend that initiating a dedicated research effort to delve into this issue is warranted.

SUMOylation is crucial for maintaining elevated transcription. Specifically, studies via SUMO proteomics have shown that the majority of SUMOylated proteins are nuclear proteins and are involved in various processes, ranging from chromosome segregation to DNA repair, DNA replication, nuclear transport, transcription, and telomere length regulation^67,68^. Importantly, impaired cellular SUMOylation has been shown to lead to a decrease in RNA Pol II levels, thereby linking SUMOylation to increased transcription^69^. Given the important role of SUMOylation in the stress response of eukaryotic cells, we hypothesize that SUMOylation is involved in the upregulation of post-traumatic inflammatory factor transcription, and in this study, we identified a modified target, BRD4, for this modification.

TRMT112 is considered a central “hub” protein that regulates the metabolic stability and enzymatic activity of at least seven MTases in mammalian cells^70^. For example, the rRNA N6-adenosine-methyltransferase (METTL5) must form a heterodimer with TRMT112 to achieve metabolic stability in cells^43^, and THUMP domain-containing protein 3 (THUMPD3) also needs to interact with TRMT112 to activate its methyltransferase activity^71^. However, to the best of our knowledge, the role of TRMT112 in inflammation has not been elucidated. In this study, we revealed for the first time the binding relationship between BRD4 and TRMT112 in inflamed synovioblasts and demonstrated the function of TRMT112 in guaranteeing the expression of BRD4-controlled inflammatory cytokines, which is related to TRMT112-mediated promotion of the stability of the transcripts of the IL-1β, TNF-α and IL-6 genes.

In summary, this study reveals the promoting effect of Cbx4-mediated BRD4 SUMOylation on the expression of inflammatory factors in PTOA. This study provides a new theoretical basis for further understanding the proinflammatory mechanisms of BRD4 and offers promising biological targets for the treatment of PTOA.

## Data Availability

All the data generated or analyzed during this study are included in this article. The datasets used and/or analyzed during the current study are available from the corresponding author upon reasonable request.
